# Variable frequency deep brain stimulation of subthalamic nucleus to improve freezing of gait in Parkinson's disease

**DOI:** 10.1093/nsr/nwae187

**Published:** 2024-06-07

**Authors:** Fumin Jia, Aparna Wagle Shukla, Wei Hu, Yu Ma, Jianguo Zhang, Leonardo Almeida, Chris Kao, Yi Guo, Shizhong Zhang, Yingqun Tao, Zhipei Ling, Xin Xu, Zhiquan Yang, Fan-gang Meng, Xinhua Wan, Hesheng Liu, Peter E Konard, Luming Li

**Affiliations:** National Engineering Laboratory for Neuromodulation, Tsinghua University, China; University of Florida Center for Movement Disorders and Neurorestoration, USA; University of Florida Center for Movement Disorders and Neurorestoration, USA; Tsinghua University Yuquan Hospital, China; Beijing Tiantan Hospital, Capital Medical University, China; University of Florida Center for Movement Disorders and Neurorestoration, USA; School of Medicine, Vanderbilt University, USA; Peking Union Medical College Hospital, China; ZhuJiang Hospital of Southern Medical University, China; The General Hospital of Shenyang Military, China; Chinese PLA General Hospital, China; Chinese PLA General Hospital, China; Xiangya Hospital Central South University, China; Beijing Neurosurgical Institute, Capital Medical University, China; Peking Union Medical College Hospital, China; Athinoula A. Martinos Center for Biomedical Imaging, Harvard Medical School, USA; School of Medicine, Vanderbilt University, USA; National Engineering Laboratory for Neuromodulation, Tsinghua University, China; Precision Medicine & Healthcare Research Center, Tsinghua-Berkeley Shenzhen Institute, China; Man-Machine-Environment Engineering Institute, School of Aerospace Engineering, Tsinghua University, China; Center of Epilepsy, Beijing Institute for Brain Disorders, China

Deep brain stimulation (DBS) of the subthalamic nucleus (STN) is a well-established therapy for patients with Parkinson's disease (PD) presenting with medication refractory tremors, motor fluctuations and dyskinesias induced by dopaminergic medications [1]. However, traditional high-frequency stimulation (HFS) does not effectively control axial symptoms such as gait dysfunction, postural instability and freezing of gait (FOG) [2,3]. We hypothesize that a novel paradigm of variable frequency stimulation (VFS) with alternating HFS and low-frequency stimulation (LFS) could benefit both axial as well as appendicular motor features when compared to conventional HFS [4]. It has been proven that these novel stimulation settings are well tolerated and freezing episodes as well as gait speed were improved in a previous case study [5]. We sought to further confirm these findings in a prospective pilot study in Tsinghua University Yuquan Hospital, Beijing, China (Ethical Approval ID: YQ 2015-08-12).

Twenty-eight PD patients with FOG (16 males and 12 females) were recruited (Fig. 1A), and written informed consent was obtained from all participants. The mean age for participants was 56.9 (±9.5) years and the mean duration after DBS surgery was 25.4 (±28.4) months (Table S1). The mean dose for dopaminergic medications at the time of study was 549.5 (±302.1) mg and the mean total electrical energy delivered (TEED) was 76.5 ± 34.9 mA^2^·Ω·Hz·μs.

**Figure 1.**
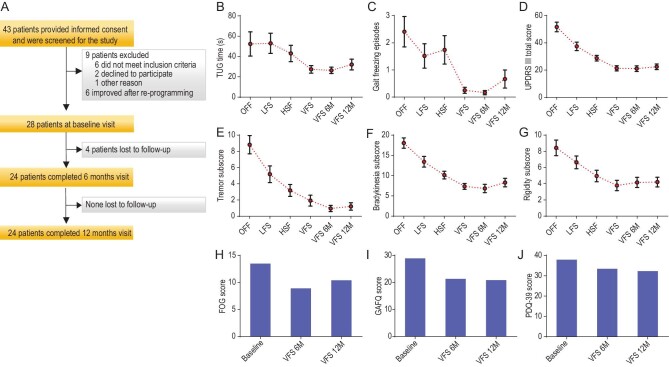
(A) Study enrollment and follow-up. (B) The mean (±SD) of timed up and go (TUG) time during DBS off, high frequency stimulation (HFS), low frequency stimulation (LFS), variable frequency stimulation (VFS), VFS at 6 months (6M) and VFS at 12 months (12M). (C) The mean (±SD) of the numbers of freezing episodes during DBS off, LFS, HFS, VFS, VFS at 6M and 12M. (D) The mean (±SD) of total motor UPDRS III scores recorded during DBS off, LFS, HFS, VFS, VFS at 6M and 12M. (E) The mean (±SD) of the tremor subscores recorded during DBS off, LFS, HFS, VFS, VFS at 6M and 12M. (F) The mean (±SD) of the bradykinesia subscores recorded during DBS off, LFS, HFS, VFS, VFS at 6M and 12M. (G) The mean (±SD) of the rigidity subscores recorded during DBS off, LFS, HFS, VFS, VFS at 6M and 12M. (H) The freezing of gait (FOG) questionnaire obtained at baseline, after 6 months of VFS programming and 12 months of programming. (I) The gait and falls questionnaire (GAFQ) at baseline, and VFS at 6M and 12M. (J) Parkinson's Disease Questionnaire (PDQ-39) at baseline, and VFS at 6M and 12M.

These PD subjects were scheduled for three study visits that were: baseline, VFS 6 months and VFS 12 months. During each study visit, subjects were required to withhold dopaminergic medications for at least 12 hours. The baseline visit included four DBS settings: HFS (130–185 Hz), DBS OFF, LFS (60–80 Hz) and VFS, and lasted for a total of 6 hours. In the VFS setting, trains of HFS interleaved by LFS that cycled every 50–300 seconds were delivered. Once the programming was accomplished, patients remained in each setting for at least 60 minutes prior to a clinical rating including a video recording for blinded clinical rating.

During DBS OFF, the mean (±SD) total time on the timed up and go (TUG) task was 52.4 (±49.3) seconds and the number of freezing episodes was 2.4 (±2.3). The statistical comparisons were performed between LFS, VFS and HFS and are presented in Table S2. In the analysis of primary outcomes, there was a significant effect of DBS frequency on the gait, including the total duration of TUG time (F_2, 25_ = 5.13, *P* = 0.005) (Fig. 1B) and the number of freezing episodes (F_2, 25_ = 5.8, *P* = 0.004) (Fig. 1C). The VFS setting resulted in significant improvement in total TUG time when compared to HFS (56.9% change, *P* = 0.005) and LFS (93.4% change, *P* = 0.005). Similarly, the number of freezing episodes was found to significantly reduce with VFS compared to HFS (>100% decrease, *P* = 0.004) and LFS (>100% decrease, *P* = 0.004).

The motor outcomes revealed that in the Wilk's Lambda test analysis, there was a significant effect of frequency on the total UPDRS score (F_2, 26_ = 20.5, *P* < 0.0001) (Fig. 1C). The *post hoc* comparisons were significant for VFS-HFS (35.2% change, *P* < 0.0001) and VFS-LFS (76.1% change, *P* < 0.0001) comparisons. Consistent with our hypothesis, we found VFS resulted in greater total UPDRS score change compared to HFS and LFS settings. Furthermore, the VFS effects on motor outcome were sustained for 6 months (t = 0.371, *P* = 0.714) and 12 months (t = −0.66, *P* = 0.52). The mean (±SD) total motor UPDRS III score recorded during DBS OFF was 51.7 (±18.5) with 9 subjects reporting inability to walk.

The secondary gait outcomes that included UPDRS items 28, 29 and 30 also revealed findings consistent with our hypothesis and VFS did significantly better than HFS and LFS. The VFS improvement was sustained for 6 and 12 months with regard to the TUG time (t = 0.47, *P* = 0.64, 6 months; t = −1.05, *P* = 0.30, 12 months) and the number of freezing episodes (t = −0.57, *P* = 0.58, 6 months; t = −1.67, *P* = 0.11, 12 months).

There was a significant effect of DBS frequency on the PD tremors (F_1, 27_ = 18.9, *P* < 0.0001) (Fig. 1D), bradykinesia (F_2, 26_ = 21.6, *P* < 0.0001) (Fig. 1E) and rigidity (F_2, 26_ = 7.9, *P* < 0.0001) (Fig. 1F). VFS DBS was found to significantly reduce tremors in the VFS-HFS comparison (68.4% change, *P* = 0.005) and VFS-LFS comparison (>100% change, *P* < 0.0001). VFS also improved bradykinesia (VFS-HFS: 36.5% change, *P* < 0.0001; VFS-LFS: 81.1% change, *P* < 0.0001) and rigidity (VFS-HFS: 31.6% change, *P* = 0.001; VFS-LFS: 73.7% change, *P* < 0.0001) as compared to HFS and LFS settings. The VFS improvements were sustained for 6 and 12 months with regard to the tremors (t = 1.86, *P* = 0.075, 6 months; t = 1.35, *P* = 0.19, 12 months) and bradykinesia (t = 1.08, *P* = 0.30, 6 months; t = −0.97, *P* = 0.35, 12 months).

The quality-of-life was noted to remain the same when the baseline study was compared with 6 and 12 months (baseline: 37.7 ± 3.9; 6 months: 33.5 ± 13.1; *P* = 0.09; 12 months: 32.3 ± 16.6; *P* = 0.08) (Fig. 1J). However, there were significant improvements on the FOGQ (baseline: 13.5 ± 4.2; 6 months: 8.9 ± 3.5, *P* = 0.0001; 12 months: 10.4 ± 4.5; *P* = 0.0001) (Fig. 1H) and the gait and falls questionnaire (GAFQ) (baseline: 28.9 ± 11.6; 6 months: 21.3 ± 8.6, *P* = 0.005; 12 months: 20.9 ± 10.7; *P* = 0.01) (Fig. 1I).

The main findings of the study were that VFS improved the gait speed and reduced the number of freezing episodes and simultaneously demonstrated significant control of tremors, bradykinesia and rigidity in PD patients with STN DBS. We also addressed the issue arising in LFS studies related to temporary control of PD symptoms where Ricchi *et al.* found that 11 STN DBS-treated PD patients had significant immediate improvement of gait without PD segmental symptoms worsening at 3 hours, by switching the stimulation frequency from 130 Hz to 80 Hz [6]. However, at 1-month and 5-month follow-up evaluations, this gait improvement was not maintained. Notably, three patients were switched back to 130 Hz because of unsatisfactory control of motor symptoms. There are several studies that suggest axial symptoms can be treated when DBS therapy is delivered at frequencies lower (usually 60–80 Hz) than the conventional HFS in the range of 130–180 Hz [7–9]. However, a major limitation of LFS is that the cardinal motor symptoms of PD, such as tremors, bradykinesia and rigidity, do not seem to respond and may sometimes worsen. In addition, there are concerns that the benefits drawn from LFS are transient [6] and many patients may have to switch back to HFS DBS [10]. In contrast with these findings, our study observed persistent improvements in gait scores along with other motor improvements lasting 12 months. Our results also found significant improvements in tremor and rigidity, which impacted the total UPDRS scores. Given that VFS was well tolerated in all studied patients, we think it is reasonable that VFS could possibly be applicable for long-term management of some STN DBS patients with intractable gait dysfunction.

There are several limitations that should be discussed. Patients were not randomized to different DBS settings, but they were blinded to the sequence of DBS settings. A wash-out period for the settings of LFS and VFS was employed to improve patient tolerance, but carryover effects between different DBS settings could not be excluded. We could not test the effects of LFS and VFS with maintenance of a constant TEED. In the case of a VFS setting, an accurate TEED could not be calculated. In addition, the effect of LFS stimulation is consistently worse than that of HFS in our study. Too short a period between HFS and LFS might be the reason. Besides that, for the parameter setting, we only changed stimulation frequency during each condition, with voltage, pulse width and electrode remaining the same during baseline investigation. Regarding the LFS setting, the use of voltage adjustments for the monopolar setting, if they are higher than 3.5 V, is not recommended. Thus, lower than half TEED in the LFS condition compared with that of HFS might also result in poor symptoms. It might be useful to maintain TEED in future studies. Finally, the longitudinal follow-up did not involve a control group and was blinded only to the clinical video raters.

Interestingly, the quality-of-life measures on the PDQ-39 were no better than with HFS. It is possible that the PDQ-39 may not have been sensitive enough to pick up changes in the subscores of the FOG and GAFQ. Additionally, the PDQ-39 can be influenced by many factors, including mood, depression and cognitive function. A future larger study powered for the PDQ-39 changes may reveal QOL improvements, but this study was not powered for such an analysis.

In conclusion, this 12-month study demonstrated that STN-VFS resulted in a significant and clinically meaningful improvement in motor symptoms. The patients who received VFS had sustained improvements in gait measures over a one-year follow-up period. This study should be followed by a larger multi-center study that is designed to reveal changes in other practical measures, such as QOL.

## Supplementary Material

nwae187_Supplemental_File
