# Supplementary material for: Variable frequency deep brain stimulation of subthalamic nucleus to improve freezing of gait in Parkinson's disease
Source: Natl Sci Rev. 2024 Jun 7;11(6):nwae187. doi: 10.1093/nsr/nwae187 (PMC11214434; doi:10.1093/nsr/nwae187)
Supplement: nwae187_Supplemental_File [file nwae187_supplemental_file.zip › table S1.docx]

Table S1 Baseline Characteristics

|  | **Patients (n=28)** |
| --- | --- |
| Age (years) | 56.9 ± 9.5 (range 37-73) |
| Male:Female | 16:12 |
| Disease duration (years) | 12.5 ± 4.7 (range 5-20) |
| Stimulation duration (months) | 25.4 ±28.4 (range 6-144) |
| Hoehn & Yahr stage | 3.3 ± 0.6 (range 2.5-5.0) |
| Levodopa daily dose equivalent (mg) | 549.5 ± 302.1 (range 0-1500) |
| Gait and falls questionnaire |  |
| Total score | 28.9 ± 10.8 (range 10-58) |
| Gait score | 21.7 ± 6.9 (range 10-38) |
| Falls score | 7.2 ± 4.4 (range 0-20) |
| FOG questionnaire | 13.5± 3.9 (range 6-24) |
| UPDRS II score | 16.6 ± 3.9 (range 7-27) |
| PDQ-39 summary index | 37.7 ± 11.3 (range 14-64) |
| PDQ-39 subscales |  |
| Mobility | 43.1 ± 17.2 (range 0-78) |
| Activities of daily living | 36.0 ± 20.1 (range 0-75) |
| Emotional well being | 35.9 ± 20.6 (range 0-75) |
| Stigma | 42.2 ± 21.6 (range 0-81) |
| Social support | 22.6 ± 15.7 (range 0-58) |
| Cognitions | 34.2 ± 17.2(range 6-63) |
| Communication | 40.2 ± 21.6 (range 0-75) |
| Bodily discomfort | 37.8 ± 24.5 (range 0-83) |
| Bilateral STN stimulation |  |
| Voltage (V) | 2.7 ± 0.5 (1.7-3.7) |
| Pulse width (μs) | 74.1 ± 11.6 (50-90) |
| Frequency (Hz) | 147.9 ± 23.8 (60-185) |
| TEED | 76.5 ± 34.9 (11.8-153.0) |

Data are mean ± SD
