# Supplementary material for: Variable frequency deep brain stimulation of subthalamic nucleus to improve freezing of gait in Parkinson's disease
Source: Natl Sci Rev. 2024 Jun 7;11(6):nwae187. doi: 10.1093/nsr/nwae187 (PMC11214434; doi:10.1093/nsr/nwae187)
Supplement: nwae187_Supplemental_File [file nwae187_supplemental_file.zip › table S2.docx]

**Table S2: Gait and Motor Assessment in different DBS settings and longitudinal follow-up data on VFS setting**

|  | |  | |  | | **Study Visit 1 Baseline** | | | |  | | |  | | **Study Visit 2/Six Month** | | | | **Study Visit 3/Twelve Month** | | | | |
| --- | --- | --- | --- | --- | --- | --- | --- | --- | --- | --- | --- | --- | --- | --- | --- | --- | --- | --- | --- | --- | --- | --- | --- |
|  | | | OFF | | VFS | | LFS | | | HFS | | |  | | VFS | | | | VFS | | | | |
|  | | | Mean  ± SD | | Mean  ± SD | | Mean  ± SD | Change^a^  % | t,  p value | | Mean  ± SD | Change^a^  % | t,  p value | ANOVA  p1value | | Mean  ± SD | Change^a^  % | t,  p value | | Mean  ± SD | Change^a^  % | t,  p value | ANOVA  p2 value |
| **TUG** |  | | | | | | | | | | | | | | | | | | | | | | |
| **Total time**  **in seconds** | | | 52.4±49.3 | | 27.4±19.6 | | 53.0±51.6 | 93.4 | t=3.043  0.005 | | 43.0±43.9 | 56.9 | t=2.787  0.005 | <0.0001 | | 26.4±15.3 | -3.6 | t=0.470  0.643 | | 32.1±25.8 | 17.2 | t=-1.053  0.303 | 0.4257 |
| **Freezing episodes** | | | 2.4±2.3 | | 0.3±0.6 | | 1.5±2.3 | >100.0 | t=3.136  0.004 | | 1.7±2.7 | >100.0 | t=3.115  0.004 | 0.0002 | | 0.2±0.4 | -33.3 | t=-0.569  0.575 | | 0.7±1.6 | >100.0 | t=-1.672  0.108 | 0.1835 |
| **UPDRSIII** |  | | | | | | | | | | | | | | | | | | | | | | |
| **Total** | | | 51.7±18.5 | | 21.3±9.9 | | 37.5±16.5 | 76.1 | t=6.729  0.0001 | | 28.8±10.9 | 35.2 | t=5.716  0.0001 | <0.0001 | | 21.1±10.2 | -0.9 | t=0.371  0.714 | | 22.5±9.9 | 5.6 | t=-0.655  0.519 | 0.7002 |
| **Gait** | | | 2.0±1.1 | | 0.9±0.7 | | 1.4±0.9 | 55.6 | t=2.867  0.008 | | 1.1±0.8 | 22.2 | t=1.652  0.110 | 0.0075 | | 0.9±0.5 | 0 | t=0.000  1.000 | | 0.9±0.7 | 0 | t=0.253  0.802 | 0.9460 |
| **Posture** | | | 2.5±1.1 | | 1.1±0.7 | | 1.9±0.8 | 72.7 | t=5.665  0.000 | | 1.6±0.8 | 45.5 | t=4.264  0.000 | <0.0001 | | 1.3±0.6 | 18.2 | t=-2.598  0.016 | | 1.2±0.8 | 9.1 | t=-1.163  0.257 | 0.0762 |
| **Postural**  **stability** | | | 2.6±0.8 | | 1.8±0.6 | | 2.1±0.5 | 16.7 | t=2.785  0.010 | | 2.0±0.4 | 11.1 | t=2.553  0.017 | 0.0052 | | 1.7±0.5 | -5.6 | t=0.700  0.491 | | 1.6±0.7 | -11.1 | t=0.720  0.479 | 0.4724 |
| **Tremor** | | | 8.8±6.0 | | 1.9±3.6 | | 5.2±5.4 | >100.0 | t=4.702  0.0001 | | 3.2±3.9 | 68.4 | t=3.047  0.005 | <0.0001 | | 1.0±1.9 | -47.4 | t=1.862  0.075 | | 1.2±2.2 | -36.8 | t=1.346  0.191 | 0.4283 |
| **Rigidity** | | | 8.4±5.1 | | 3.8±3.4 | | 6.6±4.2 | 73.7 | t=4.155  0.000 | | 5.0±3.7 | 31.6 | t=3.621  0.001 | <0.0001 | | 4.2±3.1 | 10.5 | t=-0.173  0.864 | | 4.2±2.9 | 10.5 | t=-0.277  0.784 | 0.6286 |
| **Bradykinesia** | | | 18.0±6.7 | | 7.4±3.8 | | 13.4±6.9 | 81.1 | t=6.771  0.000 | | 10.1±5.0 | 36.5 | t=4.462  0.000 | <0.0001 | | 6.8±5.0 | -8.1 | t=1.079  0.292 | | 8.3±5.1 | 12.2 | t=-0.961  0.347 | 0.2760 |

Plus–minus values are means ±SD.VFS: variable frequency stimulation; LFS: low frequency stimulation; HFS high frequency stimulation; ; UPDRS III score : Unified Parkinson's disease rating scale part III total score; TUG: Timed Up and Go Task; ^a^ Percentage change compared to VFS score at baseline;

ANOVA p1 value: A repeated measures ANOVA to compare LFS, HFS and VFS; ANOVA p2 value: A repeated measures ANOVA to compare VFS baseline, VFS 6M and VFS 12M
